# Supplementary material for: Association between vitamin D level and respiratory distress syndrome: A systematic review and meta-analysis
Source: PLoS One. 2023 Jan 26;18(1):e0279064. doi: 10.1371/journal.pone.0279064 (PMC9879443; doi:10.1371/journal.pone.0279064)
Supplement: S2 Table — (DOCX) [file pone.0279064.s002.docx]

**Supplementary Table 2: The quality assessment of included study using the Newcastle-Ottawa Scale**

| **Studies** | **Selection** | | | | **Comparability** | **Outcome** | | | **Total score** | **Overall quality** |
| --- | --- | --- | --- | --- | --- | --- | --- | --- | --- | --- |
|  | Representativeness of the exposed cohort | Selection of the non-exposed cohort | Ascertainment of exposure | Demonstration that outcome of interest was not present at start of study | Comparability of cohorts on the basis of the design or analysis | Assessment of outcome | Was follow-up long enough for outcomes to occur | Adequacy of follow up of cohorts |  |  |
| Ataseven, 2014 | * | * | * |  | ** | * | * | * | 8 | High |
| Fettah, 2015 | * | * | * |  | ** |  | * | * | 7 | High |
| Onwuneme, 2015 | * | * | * |  | * |  | * |  | 5 | Moderate |
| Yu, 2017 | * | * | * | * | * | * | * | * | 8 | High |
| Mohamed, 2018 | * |  |  |  | * | * | * | * | 5 | Moderate |
| Yang, 2018 | * | * |  | * |  |  | * |  | 4 | Moderate |
| Boskabadi, 2018 | * | * |  | * | ** |  | * | * | 7 | High |
| Kazzi, 2018 | * | * | * |  |  |  | * | * | 5 | Moderate |
| Kim, 2019 | * | * | * | * | * | * | * | * | 8 | High |
| Treiber, 2020 | * | * | * |  |  |  | * |  | 4 | Moderate |
| Ardastani, 2020 | * | * | * |  | ** | * | * |  | 7 | High |
| Matejek, 2020 | * | * | * |  | * | * | * | * | 7 | High |
| Al-Beltagi, 2020 | * | * |  | * |  |  | * | * | 5 | Moderate |
| Dogan, 2020 | * | * | * |  | ** | * | * |  | 7 | High |
| Zhang, 2021 | * | * | * |  | ** | * | * |  | 7 | High |
