# Supplementary figures and images for: Association between vitamin D level and respiratory distress syndrome: A systematic review and meta-analysis
Source: PLoS One. 2023 Jan 26;18(1):e0279064. doi: 10.1371/journal.pone.0279064 (PMC9879443; doi:10.1371/journal.pone.0279064)

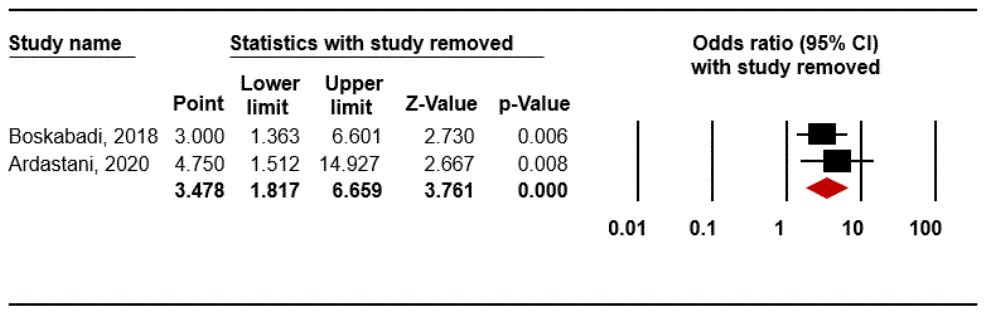

Supplement: S1 Fig — (ZIP) [file pone.0279064.s003.zip › S1-1 Fig.gif]

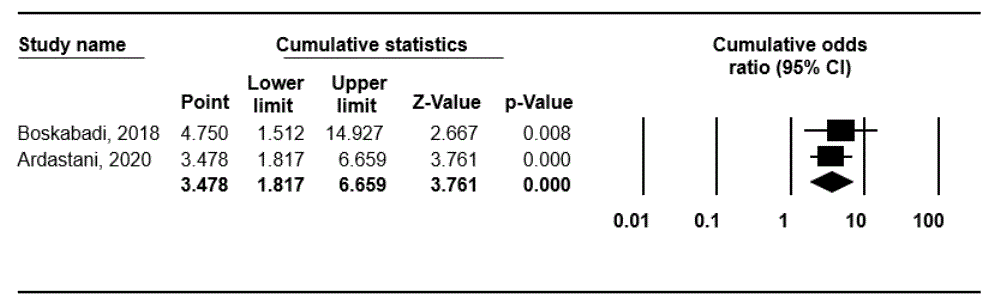

Supplement: S1 Fig — (ZIP) [file pone.0279064.s003.zip › S1-2 Fig.gif]

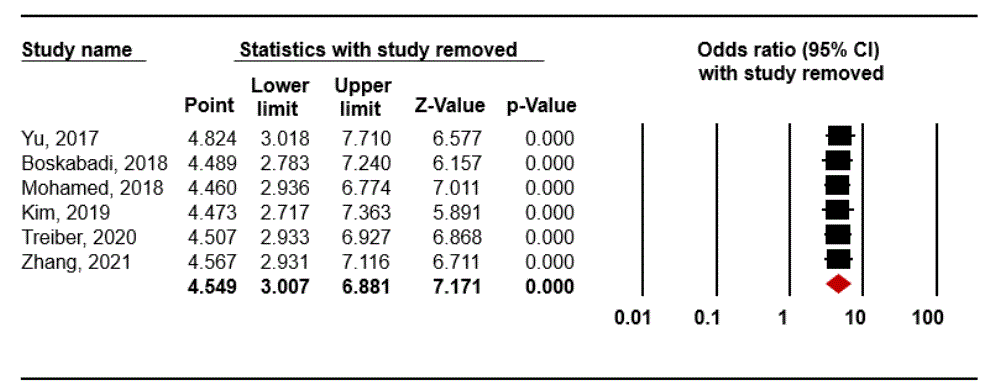

Supplement: S2 Fig — (ZIP) [file pone.0279064.s004.zip › S2-1 Fig.gif]

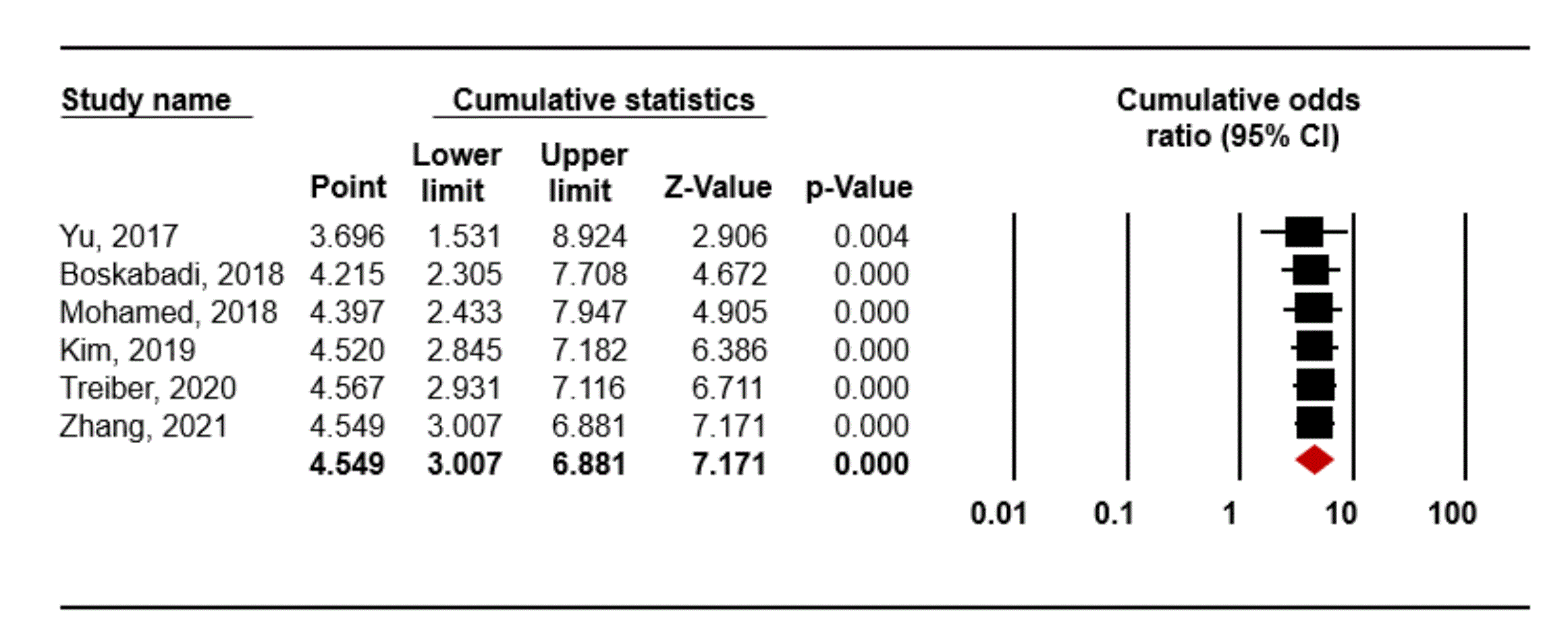

Supplement: S2 Fig — (ZIP) [file pone.0279064.s004.zip › S2-2 Fig.gif]

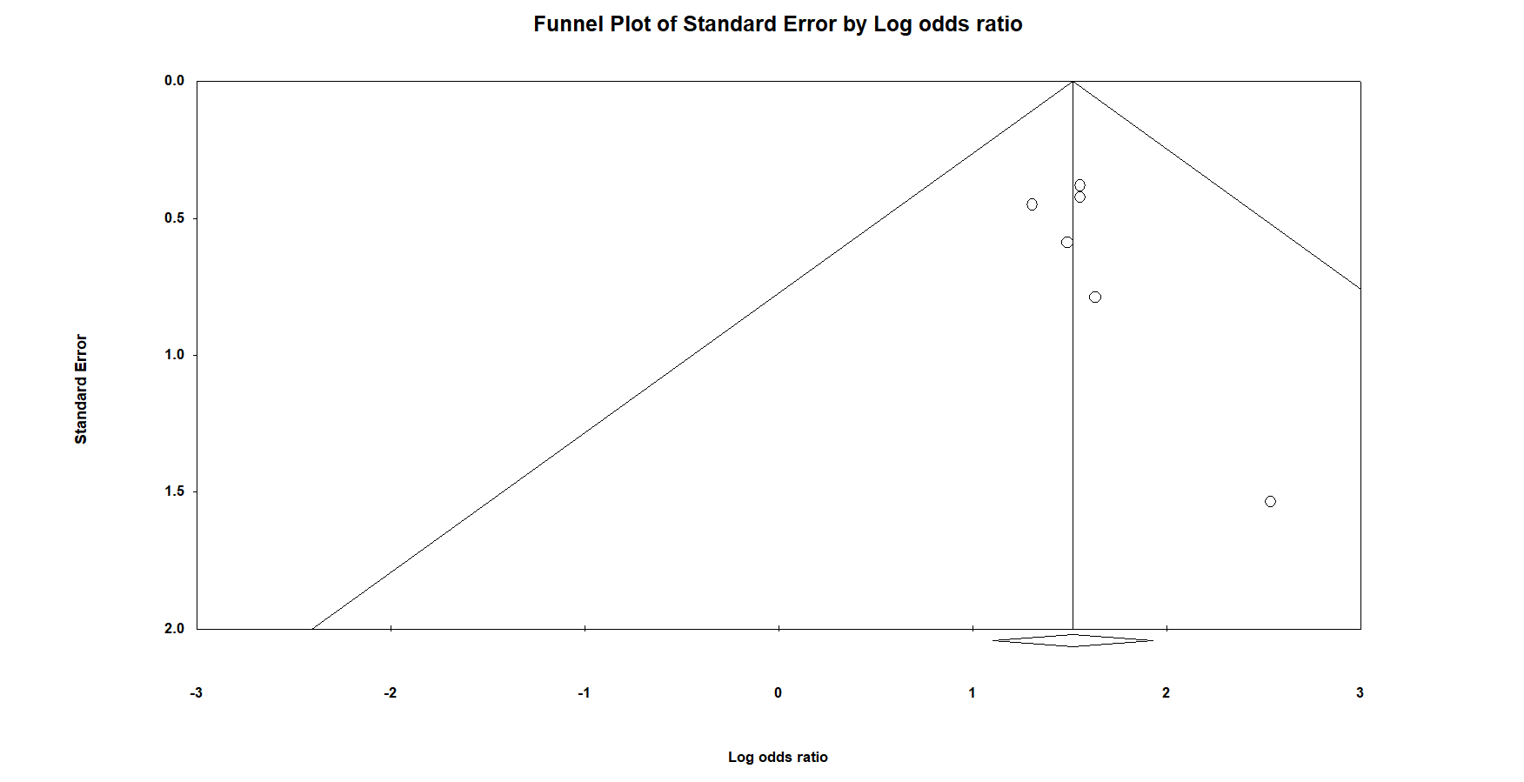

Supplement: S2 Fig — (ZIP) [file pone.0279064.s004.zip › S2-3 Fig.gif]

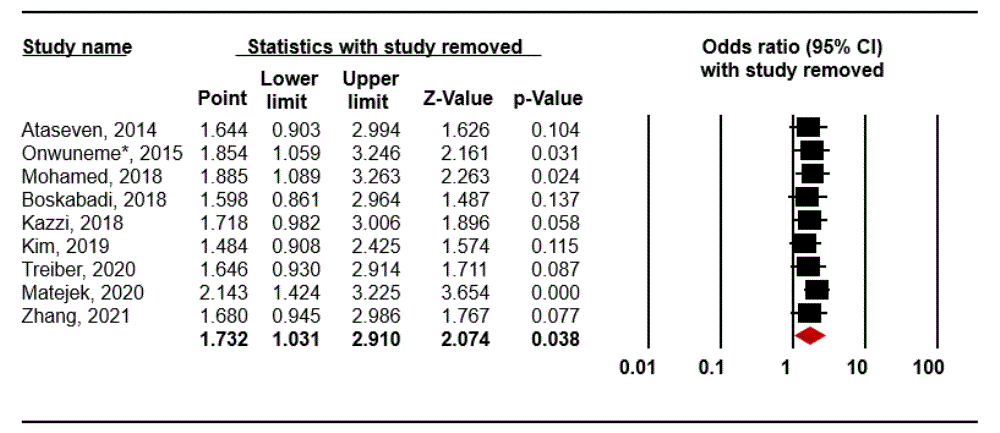

Supplement: S3 Fig — (ZIP) [file pone.0279064.s005.zip › S3-1 Fig.gif]

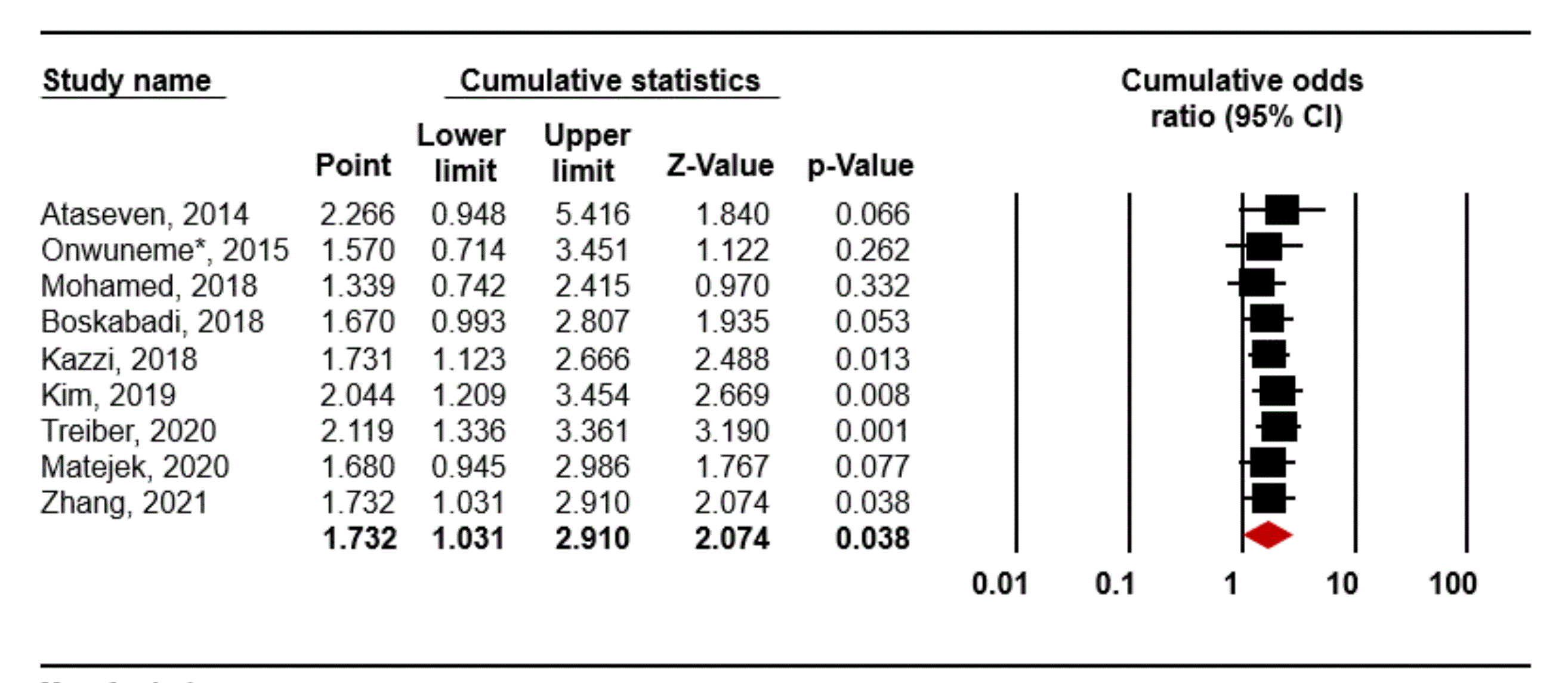

Supplement: S3 Fig — (ZIP) [file pone.0279064.s005.zip › S3-2 Fig.gif]

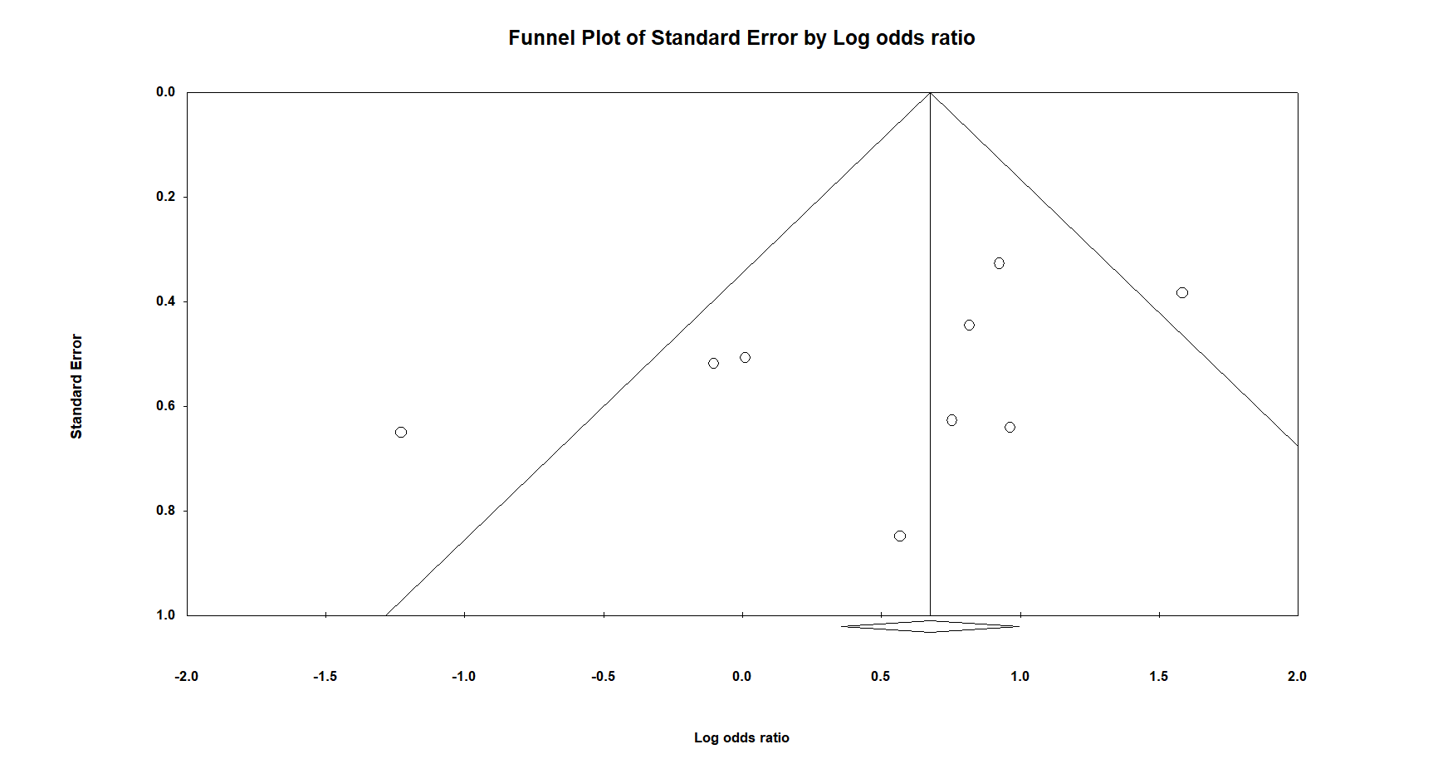

Supplement: S3 Fig — (ZIP) [file pone.0279064.s005.zip › S3-3 Fig .gif]

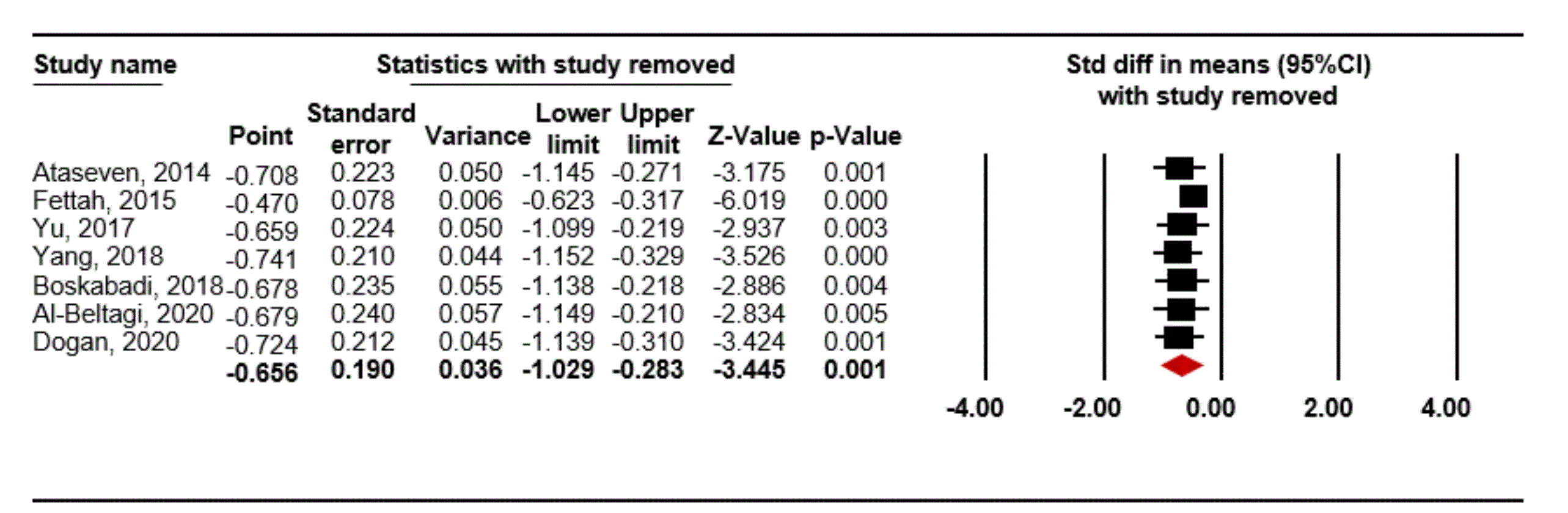

Supplement: S4 Fig — (ZIP) [file pone.0279064.s006.zip › S4-1 Fig.gif]

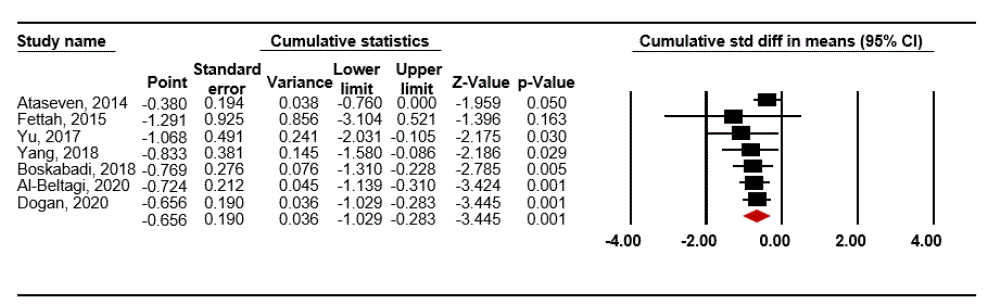

Supplement: S4 Fig — (ZIP) [file pone.0279064.s006.zip › S4-2 Fig.gif]

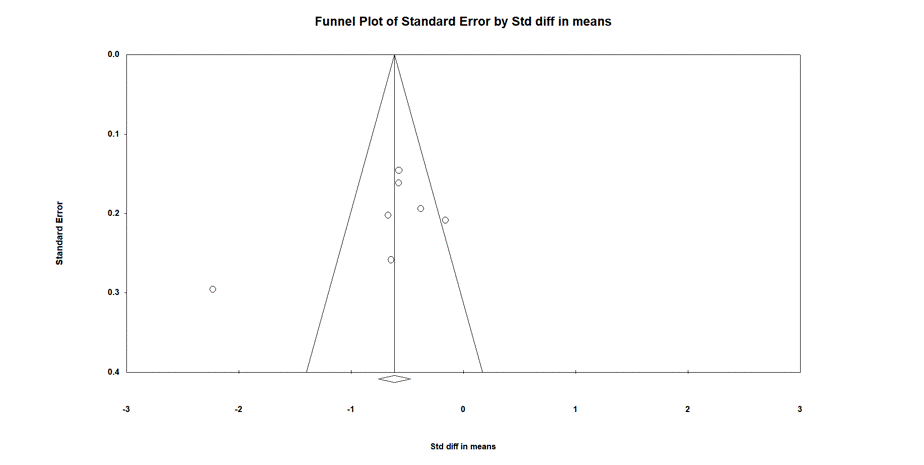

Supplement: S4 Fig — (ZIP) [file pone.0279064.s006.zip › S4-3 Fig.gif]
