# Supplementary material for: Association between vitamin D level and respiratory distress syndrome: A systematic review and meta-analysis
Source: PLoS One. 2023 Jan 26;18(1):e0279064. doi: 10.1371/journal.pone.0279064 (PMC9879443; doi:10.1371/journal.pone.0279064)
Supplement: Attachment — Submitted filename: My review_130922.pdf [file pone.0279064.s007.pdf]

Thank you to the reviewers for this interesting study with results that can be applied to clinical practice. Overall, I couldn't identify major changes that are needed.

Abstract

I believe it can be improved to synthesise the study better. Remember that most people will read the full text only if the abstract is interesting enough to call their attention.

For example, some phrases from the introduction are better written than from the abstract and could be used to improve it. I also suggest adding that authors performed the meta analysis model the reviewers used to synthesise the data, instead of citing which software they had used.

In addition to that, I believe that the PRISMA 2020 checklist for abstracts could be used as a guide to improve the abstract.

https://prisma-statement.org/documents/PRISMA_2020_abstract_checklist.pdf

Page MJ, McKenzie JE, Bossuyt PM, Boutron I, Hoffmann TC, Mulrow CD, et al. The PRISMA 2020 statement: an updated guideline for reporting systematic reviews. BMJ 2021;372:n71. doi: 10.1136/bmj.n71

Introduction

The authors wrote "The level of vitamin D within 24 h of birth is associated with the risk of BPD in preterm infants [3]", what gives an impression that this is well established in the literature, followed by: "Previous studies raised the possibility that the risk of respiratory distress syndrome (RDS) is increased in vitamin D-deficient preterm neonates, but the clinical relevance remains uncertain." showing that this matter is controversial. Please, could the authors correct this disagreement? Also, could please authors provide citations to support the last quote?

Results: Text and Figure 1:

1. Manual search - Could the authors please provide details about it? Does it mean that they searched the references?

2. In the flow chart (Fig 1) it is described that 2 articles were excluded due to "inadequate data for analysis" and 12 because "irrelevant data for analysis". However it was described in the methods that inclusion and exclusion criteria were based on design of the study, not on outcomes. So, please clarify that in the text.

3. Regarding the 2 papers that had "inadequate data for analysis", were they incomplete? Did the reviewers try to contact the study authors?

4. Besides that, the primary outcome isn't clear in the text, although one can infer that it is "respiratory distress syndrome". It would be reasonable to state it clearly in the text, as the authors could study other outcomes linked to that (like death or need of surfactant treatment, for example).

Characteristics of included studies

Regarding weight and gestational age, besides the mean, could authors please provide either standard deviation or the range for those data? They are relevant for clinicians reading the paper.

Table 1

I really like how the authors summarised the data in this table. But I have two minor suggestions

1. "Newcastle-Ottawa Scale for case-control study was used for study quality assessment, <u>others used one for cohort study</u>" - I couldn't understand this phrase, could you please rephrase it?
2. In the line explaining the study of Kim et. al, could you please correct the format of the column with vitamin D deficiency definition? So it is in the same pattern as the others.

Discussion:

BPD is used in the text, but the authors didn't make it clear that it refers to Bronchopulmonary dysplasia. Please add it on page 1, when the term first appears.

After reading the paper, two potential confusion biases still were not clear to me.

1. Influence of the gestational age - As the authors stated: "The incidence of RDS decreases with increasing gestational age at birth, from 97% at 23 weeks of gestation to 65% at 28 weeks [42], 10.5% at 34 weeks, and 0.3% at 38 weeks of gestation". Thus, we know that gestational age itself can be a protective factor for developing RDS. Did the reviewers perform any subgroup analysis regarding gestational age? Would it be possible with the data available? Could vitamin D deficiency play different roles in different patient subsets?

2. Corticoid administration during pregnancy -there is evidence showing that corticosteroid administration in preterm pregnancies can result in lower incidence of RDS. Did the studies included in the meta analysis have controlled this factor? Did the reviewers consider that while doing the analysis?

I believe both biases are relevant and should - at least - be cited in the discussion.
